# Lrrk2 R1628P variant is a risk factor for essential tremor

**DOI:** 10.1038/srep09029

**Published:** 2015-03-12

**Authors:** Yin Xia Chao, Ebonne Yulin Ng, Louis Tan, Kumar M. Prakash, Wing-Lok Au, Yi Zhao, Eng-King Tan

**Affiliations:** 1 Departments of Neurology, Singapore General Hospital; 2 Clinical Research, Singapore General Hospital; 3 National Neuroscience Institute; 4 Duke-NUS Graduate Medical School

## Abstract

Essential tremor (ET) and Parkinson's disease (PD) are two of the most common adult onset movement disorders with overlapping clinical features. PD patients with leucine-rich repeat kinase-2 (*LRRK2*) mutations may present initially with an ET phenotype. To address the possibility of a common genetic link between ET and PD, we examined the association between a common *LRRK2* R1628P gene variant and ET. The *LRRK2* R1628P was genotyped in ET cases and matched healthy controls. A total of 1277 subjects comprising of 450 ET cases and 827 controls were included. There were 40 heterozygote (GG to CG) variant out of 450 ET cases (genotypic frequency 8.9%) and 36 heterozygote variant (GG to CG, genotypic frequency 4.3%) and one homozygote variant (GG to CC) out of 827 controls. Subjects carrying the R1628P variant had a twofold increased risk of ET (p = 0.0035, OR = 2.20 and 95% confidence interval is 1.30–3.73). Using a case control methodology, we demonstrated an association between a known PD risk variant, *LRRK2* R1628P, with ET. Subjects carrying the R1628P variant had twice the risk of developing ET. The sharing of a similar gene risk variant suggests a possible pathophysiologic link between PD and ET.

Essential tremor (ET) and Parkinson's disease (PD) are two of the most common adult onset movement disorders with overlapping clinical features^1^. PD patients with leucine-rich repeat kinase-2 (*LRRK2*) mutations may present initially with an ET phenotype, suggesting a possible etiologic link^2^. *LRRK2* R1628P variant is a well-recognized PD risk factor in Asian populations^3^. To address the possibility of a common genetic link between ET and PD, we examined the association between *LRRK2* R1628P and ET.

## Results

A total of 1277 subjects comprising of 450 ET cases and 827 controls were included. The median age of ET patients was 55 years (ranging from 2 years to 82 years), and 54 years for controls (ranging from 24 years to 92 years) (p = 0.4461, Table 1). The median onset age for ET was 40 years (n = 411, p < 0.0001). The sex ratio of ET patients (251male/199 female) was similar to the sex ratio of controls (422 male/405 female, P = 0.1132, Table 1). Among the 450 cases, 161 (36%) have confirmed family history of ET.

There were 40 heterozygote (GG to CG) variant out of 450 ET cases (genotypic frequency 8.9%) and 36 heterozygote variant (GG to CG, genotypic frequency 4.3%) and 1 homozygous variant (GG to CC) out of 827 controls. Subjects carrying the R1628P variant had twice the risk of developing ET (p = 0.0035, OR = 2.20 and 95% confidence interval is 1.30–3.73) (Table 2). This association was similarly observed if we analysed only pure ethnic Chinese subjects (about 90% of our cohort).

## Discussion

Mutations in the *LRRK2* gene are associated with familial and sporadic PD worldwide. *LRRK2* c.4883G>C (R1628P) is a polymorphic risk variant for PD in ethnic Chinese populations^3^. The phenotype of R1628P carriers is similar to late-onset L-dopa-responsive PD. Here, we demonstrated for the first time that this variant doubles the risk for ET in our cohort. While the pathophysiology of ET has been debated, some *LRRK2* mutations have been known to cause PD via a toxic gain of function^5^. The mechanistic role of R1628P variant in PD is still unclear. R1628P is located in the COR domain. It is possible that a replacement of a highly basic polar arginine with a neutral non-proline causes a conformational change in the LRRK2 protein that might affect its function or the interaction with its substrates. Unlike the pathogenic mutation G2019S, it is not known if R1628P causes a toxic gain of function via an increased kinase activity. Animal models of R1628P would provide useful insights.

The increased risk of the PD variant in ET patients is interesting. It is possible that this is related to clinical heterogeneity associated with ET. Some of our ET patients may develop PD subsequently and the risk associated with the variant may be primarily attributed to this subset of ET/PD patients. Alternatively, it is also possible that the variant is directly linked to the same pathophysiologic pathways implicated in both ET and PD. To address these issues, longitudinal clinical and functional imaging follow up of ET patients with *LRRK2* R1628P variant to monitor for evidence of late-onset PD will provide useful clues.

PD patients were more likely to have a prior diagnosis of ET compared to healthy controls^6^, suggesting that both conditions can co-exist^6^,^7^. There are also genetic links between PD and ET. Alpha synuclein (a familial-linked PD gene) promoter polymorphism has been associated with both ET and PD^8^. More recently, variants of leucine-rich repeat and Ig domain containing one gene (*LINGO1*) and leucine-rich repeat and Ig domain containing two genes (*LINGO2*) have been shown to modulate risk in both conditions^9^,^10^. ET risk might be overestimated even though in clinical setting, PD and ET may be similar in some cases, but the disease concept seems different in general. ET risk may include PD risk for Lrrk2 variant R1628P. Hence, identifying “pure” ET and coexistent PD and ET may help for risk stratification and a better understanding the pathophysiologic links of the 2 conditions and potentially lead to the development of new therapeutic strategies.

The clinical and genetic heterogeneity among ET patients and incomplete penetrance are important caveats to consider in the interpretation of both linkage analysis and genetic association studies. Rarely, some other neurodegenerative or genetic diseases can present with an ET phenotype. The contribution of environmental factors and gene-environmental interaction in ET can be difficult to assess.

In conclusion, using a case control methodology, we demonstrated for the first time an association between a known PD risk variant, *LRRK2* R1628P, with ET. Subjects carrying the R1628P variant had twice the risk of developing ET. The sharing of a similar gene risk variant suggests a possible pathophysiologic link between PD and ET. Further functional studies of this variant may unravel new etiologic clues for the two conditions.

## Methods

Consecutive patients with ET who presented to tertiary referral centres and examined by movement disorders neurologists were included. Patients satisfied the diagnostic criteria of classic ET based on the recommendations of the Consensus Statement of the Movement Disorders Society^4^. Healthy individuals of similar gender, race and age were included as controls. Subjects with evidence of other neurodegenerative diseases were excluded. ET cases did not have evidence of thyroid dysfunction. Written informed consent from all the study subjects was obtained.

Genomic DNA was extracted from blood samples and *LRRK2* c.4883G>C (R1628P; rs33949390) was genotyped by Taqman SNP genotyping (Life Technologies, Singapore). All positives and selected negatives were further confirmed by capillary/Sanger sequencing analysis with standard protocols. Our study was approved by the SingHealth Centralised Institutional Review Board (CIRB) and all the methods were carried out in accordance with the approved guidelines. Fisher's Exact Test for Count Data was used to compare the categorical and numerical variables. Statistical significance was defined at p < 0.05.

## Author Contributions

All authors fulfil the criteria of authorship and no one else who fulfils the criteria has been excluded. Y.X.C., E.Y.N. and E.K.T. had the idea, jointly designed the experiment and performed data analysis. L.T., K.M.P., W.L.A. and E.K.T. conducted clinical assessments. Y.X.C. and E.K.T. were involved in sample collection. E.Y.N. and Y.Z. performed genetic analysis. All authors were involved in writing and critically revising the article, and all have approved the final submitted version. E.K.T. accepts full responsibility for the work and controlled the decision to publish. All authors read and approved the final manuscript.

## Figures and Tables

**Table 1:** Demographics of controls and ET cases

	Controls	ET	P value
**Age**	54 (24, 92)	55 (15, 86)	0.4461
**Gender**			
**Male/Female**	422/405 (1.04)	251/199(1.26)	0.1132

**Table 2:** Genotype of Lrrk2 R1628P in ET cases and controls

Genotype	Controls	ET	OR(95%CI)	P value
**GG**	790	410	Reference	
**CG**	36	40	2.28 (1.34, 3.88)	0.0065
**CC**	1	0	0	
**CC + CG**	37	40	2.20 (1.30, 3.73)	0.0035
